# Altered dynamic functional connectivity of auditory cortex and medial geniculate nucleus in first-episode, drug-naïve schizophrenia patients with and without auditory verbal hallucinations

**DOI:** 10.3389/fpsyt.2022.963634

**Published:** 2022-09-07

**Authors:** Kangkang Xue, Jingli Chen, Yarui Wei, Yuan Chen, Shaoqiang Han, Caihong Wang, Yong Zhang, Xueqin Song, Jingliang Cheng

**Affiliations:** ^1^Department of Magnetic Resonance Imaging, The First Affiliated Hospital of Zhengzhou University, Zhengzhou, China; ^2^Department of Psychiatry, The First Affiliated Hospital of Zhengzhou University, Zhengzhou, China

**Keywords:** schizophrenia, auditory verbal hallucination, auditory cortex, medial geniculate nucleus, dynamic functional connectivity

## Abstract

**Background and objective:**

As a key feature of schizophrenia, auditory verbal hallucination (AVH) is causing concern. Altered dynamic functional connectivity (dFC) patterns involving in auditory related regions were rarely reported in schizophrenia patients with AVH. The goal of this research was to find out the dFC abnormalities of auditory related regions in first-episode, drug-naïve schizophrenia patients with and without AVH using resting state functional magnetic resonance imaging (rs-fMRI).

**Methods:**

A total of 107 schizophrenia patients with AVH, 85 schizophrenia patients without AVH (NAVH) underwent rs-fMRI examinations, and 104 healthy controls (HC) were matched. Seed-based dFC of the primary auditory cortex (Heschl's gyrus, HES), auditory association cortex (AAC, including Brodmann's areas 22 and 42), and medial geniculate nucleus (MGN) was conducted to build a whole-brain dFC diagram, then inter group comparison and correlation analysis were performed.

**Results:**

In comparison to the NAVH and HC groups, the AVH group showed increased dFC from left ACC to the right middle temporal gyrus and right middle occipital gyrus, decreased dFC from left HES to the left superior occipital gyrus, left cuneus gyrus, left precuneus gyrus, decreased dFC from right HES to the posterior cingulate gyrus, and decreased dFC from left MGN to the bilateral calcarine gyrus, bilateral cuneus gyrus, bilateral lingual gyrus. The Auditory Hallucination Rating Scale (AHRS) was significantly positively correlated with the dFC values of cluster 1 (bilateral calcarine gyrus, cuneus gyrus, lingual gyrus, superior occipital gyrus, precuneus gyrus, and posterior cingulate gyrus) using left AAC seed, cluster 2 (right middle temporal gyrus and right middle occipital gyrus) using left AAC seed, cluster 1 (bilateral calcarine gyrus, cuneus gyrus, lingual gyrus, superior occipital gyrus, precuneus gyrus and posterior cingulate gyrus) using right AAC seed and cluster 2 (posterior cingulate gyrus) using right HES seed in the AVH group. In both AVH and NAVH groups, a significantly negative correlation is also found between the dFC values of cluster 2 (posterior cingulate gyrus) using the right HES seed and the PANSS negative sub-scores.

**Conclusions:**

The present findings demonstrate that schizophrenia patients with AVH showed multiple abnormal dFC regions using auditory related cortex and nucleus as seeds, particularly involving the occipital lobe, default mode network (DMN), and middle temporal lobe, implying that the different dFC patterns of auditory related areas could provide a neurological mechanism of AVH in schizophrenia.

## Introduction

Schizophrenia is a kind of serious mental illness marked by hallucinations, delusions, and behavioral problems (1, 2), which is also described as a “disconnection syndrome” (3). With a prevalence of 60–90%, AVH is a major pathological feature of schizophrenia (4). Schizophrenia patients with AVH can vividly perceive sound without external real acoustic stimulation. The symptoms of AVH can lead to increased stress and dysfunction, and they are even linked to an increased risk of suicide (5). AVH persists after antipsychotic medication treatment in approximately 1/3 of schizophrenia patients with AVH (6, 7). Despite much research focusing on the mechanism of AVH, the results remained inconsistent. Therefore, the underlying neural mechanism of AVH is essential, which can improve our understanding of schizophrenia and guide more specific treatments (8–11).

Various neuropathological mechanism models of AVH have been proposed, among them, the verbal self-monitoring (VSM) model (12, 13) and auditory-related regions activations model (14) are the most concerned. Self-monitoring deficits in schizophrenia, according to the VSM model, are caused by abnormal brain integration impairing the communication of the associative discharge (15, 16). AVH is thought to be caused by aberrant functional connections between auditory and language-related brain areas, and these connectivity are strongly linked to the degree of AVH (17–22). There's also proof of aberrant static functional connectivity (sFC) in the auditory cortex, which is linked to default mode network (DMN) (21, 22). Alderson et al. (4) summarize that sFC in the left superior temporal gyrus is disrupted in schizophrenia patients with AVH and DMN appears atypical connectivity with other resting state networks (RSNs). According to the model of auditory-related regions activations, the abnormal self-activation of the auditory cortex generates auditory signals and transmits them to other areas even without external auditory stimulus, so as to appear auditory hallucinations in patients. Elevated activity in auditory-related areas is linked to AVH, Dierks et al. (23) found that the Broca area and primary auditory cortex were activated when auditory hallucination occurred by comparing the brain activities of the same patient with and without auditory hallucination at rest. Hunter et al. (24) also reported that the auditory cortex was abnormally activated when auditory hallucinations occurred through the study of patients with AVH without mental diseases. Taken together, whether abnormal spontaneous neural activity or connectivity with other regions, the auditory-related regions is of great significance to the AVH.

Auditory-related brain regions include several temporal gyri and nuclei. Combined with the electrophysiological research on primate auditory system and the functional imaging study of human auditory perception, the researchers propose that the sound is first received and preliminarily processed by the primary auditory cortex (Heschl's gyrus, HES), then transmitted to auditory association cortex (AAC, located in Brodmann's areas 22 and 42) for processing, and then transmitted to the higher information processing brain areas through the thalamus (mainly located in the medial geniculate nucleus, MGN) (25–28). Up to now, multiple studies have revealed abnormal activation or functional connectivity of auditory-related brain regions in schizophrenia patients with auditory hallucinations. Previous work has revealed that MGN, AAC and HES have been associated with AVH in schizophrenia (29, 30). Therefore, the study taking HES, AAC, and MGN as seeds will reveal the abnormal connections of auditory-related brain regions in schizophrenic patients with AVH, so as to verify the above hypothesis.

So far, the majority of fMRI studies investigate the mechanism of AVH in schizophrenia by using the method of sFC, with the assumption that FC between brain regions is constant across the scanning time. However, a growing number of evidence shows the considerable fluctuations in rs-fMRI FC over time. Consequently, as an emerging approach, dynamic FC has been increasingly focused on by recent fMRI research to detect dynamic connectivity patterns throughout the scanning period, and reflect the dynamic characteristics of interregional communication (31–35). Dynamic functional connectivity has been successfully used to distinguish schizophrenia from other mental diseases (36) and classify schizophrenia (37, 38). So far, only a few researchers study the auditory hallucination mechanism of schizophrenia using the dFC method. Some studies underly the whole brain dynamic patterns with ICA, Zhang et al. (39) have manifested that schizophrenia patients with AVH switched among several states using the dFC method, with the aberrant connectivity of language-related areas. However, there is no study on the dFC of the auditory-related regions in schizophrenia with AVH.

In our study, first-episode and drug- naïve schizophrenia patients with and without AVH as well as HCs were recruited. We first investigated voxel-wise dFC between the auditory-related regions (AAC, HES, and MGN) and the whole brain in three groups; Furthermore, in schizophrenia patients with AVH, we analyze the correlation between changed dFC values and symptom indicators. We assumed that schizophrenia patients with AVH may present more or less dynamic FC fluctuation of auditory-related regions throughout scanning duration compared to NAVH and HC groups, which could allow us to understand more about the pathophysiological processes of AVH in schizophrenia.

## Materials and methods

### Participants

The present study collected 107 schizophrenia patients with AVH, 85 without AVH(NAVH), and 104 healthy controls (HC) matched by age and sex were recruited for this study. According to the Diagnostic and Statistical Manual of Mental Disorders, Fourth Edition (DSM-IV), two well-trained clinical psychiatrists confirmed the patients' diagnosis of schizophrenia. The patients had never got drug, physical therapy, or counseling, and the disorder had lasted no more than 3 years. The symptoms were assessed by the Positive and Negative Symptom Scale (PANSS), and the degree of AVH was evaluated by the Auditory Hallucination Rating Scale (AHRS). The questionnaire assessment of patients was completed by two investigators after training. Patients in the AVH group encountered AVH in the last 4 weeks, with the majority occurring in the previous week, whereas patients in the NAVH group had no AVH during their entire lifespan or in the previous 4 months.

The present study was approved by the Ethics Committee of the First Affiliated Hospital of Zhengzhou University. Participants in this study need to meet the following inclusion criteria: (1) All patients were diagnosed according to Diagnostic and Statistical Manual of Mental Disorders, Fourth Edition (DSM-IV), (2) First onset, without any treatment such as medication, psychotherapy, and electric shock, (3) Han nationality, right-handed, (4) The illness duration of all patients was < 3 years, and the diapause was < 6 months. Participants with the following conditions will be excluded from this study: (1) a history of head trauma or serious physical impairment, (2) drug abuse or alcohol addiction, (3) psychiatric illness produced by physical diseases, (4) pregnancy or any contraindications for MRI. Any neurological or psychiatric condition, as well as related family history, are exclusion factors for the HC group. Following a thorough explanation of the study, all participants completed informed consent.

### Data acquisition

The magnetic resonance imaging (MRI) was carried out on a 3.0 T MRI scanner, and an 8-channel receiver array head coil (GE, USA) was used during scanning. All subjects were asked to lie still with their eyes closed and remain aware while using the foam cushioning. During scanning, earplugs were utilized to reduce strepitus impact.

The magnetic resonance imaging (MRI) was performed on a 3.0 T MRI scanner (Discovery MR750, GE, USA) with an 8-channel receiver array head coil. Participants were required to lie still using the foam padding and remain alert with their eyes closed without thinking anything. Earplugs were used to reduce the interference of noise during scanning. Structural images were acquired with a 3D T1 BRAVO sequence, the following sequence parameters were applied: repetition time (TR)/echo time (TE) = 8.2/3.2 ms, slices = 188, slice thickness = 1 mm, slice gap = 0 mm, flip angle (FA) = 12°, field of view (FOV) = 25.6 × 25.6 cm^2^, number of Averages = 1, data matrix = 256 × 256, voxel size = 1 × 1 × 1 mm^3^, scan time = 4.33 min. The following settings were used to acquire functional images transversely with a gradient spin echo planar imaging sequence: TR/TE = 2000/30 ms, slices = 32, slice thickness = 4 mm, slice gap = 0.5 mm, FA = 90°, field of view (FOV) = 22 × 22 cm^2^, number of averages = 1, data matrix = 64 × 64, voxel size = 3.4375 × 3.4375 × 4 mm^3^, and 180 volumes lasting for 360 sec.

### Data preprocessing

The resting state functional images preprocessing was preprocessed using DPABI toolbox (http://rfmri.org/dpabi) based on MATLAB (MathWorks). The first five volumes, which allowed participants to become adjusted to scanning surroundings, were eliminated. Then, further preprocessing was performed: (1) slice timing, (2) realignment in order to correct head motion, participants with a head motion > 3 mm or a 3° rotation will be excluded from the study, (3) normalization, the DARTEL method was applied to conduct normalization. Individual structural images were initially co-registered to the mean functional image, after which the modified structural images were segmented and normalized to MNI space (the resampled voxel size of 3 × 3 × 3 mm^3^), (4) detrending, (5) temporal band-pass filtering (0.01–0.08 Hz); (6) head motion parameters, the averaged white matter signal, and the cerebrospinal fluid signal were all regressed out of the confounding signals (Friston-24).

### Definition of region of interest

We selected 6 regions of interest (ROIs) to analyze whole-brain dFC, including bilateral HES, ACC, and MGN. The HES gyrus and medial geniculate (MGN) were selected as the ROIs for dFC analysis on the basis of the automated anatomical labeling atlas 3 (40). The Brodmann atlas was selected in the software WFU_PickAtlas (41) to produce the ROI of AAC, involving the combination of the Brodmann's areas 22 and 42.

### Dynamic FC analyses

Sliding time-window analysis can explain the variance of dFC, which can be defined as the temporal features of FC during the scan. This method was adopted to generate dFC maps for each participant using DPABI toolbox (42). Previous studies have shown that the suitable length for capturing dynamic FC mode ranges from 30 sec to 1 min when using Hamming window for time window method (32). The sliding window's length was chosen to walk a fine line among detecting fast changing dynamic linkages with a shorter window and accurately estimating related activities over wider window sections. Finally, 30 TR width (60s) and 1 TR step was applied to this study, in which the time series of each participant were divided into 30 TR windows. In each window, we applied seed-based functional connectivity analysis, and within the entire brain, by computing the correlation of average time course between the seeds and all other voxels, the FC maps of all ROIs were acquired. Then, zFC maps were generated *via* Fisher's z-transformation. Finally, we calculated all voxels' dFC values through computing the standard deviation of zFC values over windows.

### Statistical analysis

Statistical Package for the Social Sciences (SPSS) version 26.0 was used to analyze the demographic and clinical data. One-way analyses of variance (ANOVA) were used for age and years of education to estimate the differences among the AVH, NAVH, and HC groups. A chi-square test was used for gender comparisons among groups. Between AVH group and NAVH group, two sample *t*-tests were applied to estimate the difference in PANSS and AHRS scores. Statistical significance was all determined by *p* < 0.05.

ANOVA was performed to examine the differences in dFC of each ROI to all voxels in the entire brain among three groups (AVH, NAVH, and HC), with age, gender, years of education, and head motion included as covariates. Using DPABI toolbox, we applied the Gaussian random field (GRF) method for multiple comparison correction (voxel-wise *p* < 0.005, cluster-wise *p* < 0.05). The dFC values of clusters which is statistically significant were extracted separately to conduct *post hoc* pairwise comparisons (*p* < 0.05, Bonferroni correction).

We analyzed the correlation between the extracted dFC values of significant clusters (among AVH, NAVH and HC groups) and PANSS scores. In addition, we also analyzed the correlation between the extracted dFC values of significant clusters (between AVH and NAVH group) and the AHRS scores of AVH group. Spearman's correlation analyses were performed using SPSS with Bonferroni correction (*p* < 0.05).

### Validation analysis

In the dynamic FC analysis by sliding window approach, the window width is an important characteristic. To confirm the dFC variability results in this study, aside from 30 TR, we also ran validation tests on other sliding window lengths. We reconstructed the dFC maps for each ROI by a window length of 50 and 60 TRs. Besides, between the AVH and NAVH groups, the dFC results were reanalyzed.

## Results

### Clinical and demographic characteristics

There were no significant differences in age, gender, and educational level between the AVH, NAVH, and HC groups. Between the AVH and the NAVH groups, there was no significant difference in PANSS total score, positive, negative, and general scores (Table 1).

**Table 1:** Demographic and clinical data of AVH, NAVH, and HC groups [Mean (SD)].

	**AVH (*n* = 107)**	**NAVH** ** (*n* = 85)**	**HC (*n* = 104)**	***F/χ^2^/t***	***P***
Age (yeas)	21.50 ± 8.33	23.60 ± 8.67	22.40 ± 5.53	1.814	0.165
Sex (male/female)	42/65	46/39	50/54	4.343	0.114
Education (years)	10.80 ± 2.84	11.19 ± 3.26	11.39 ± 3.33	0.957	0.385
AHRS	22.59 ± 1.15	-	-	-	-
PNASS					
Positive	20.79 ± 5.60	20.25 ± 5.99	-	0.652	0.515
Negative	21.21 ± 6.26	22.53 ± 6.67	-	1.404	0.162
General	41.83 ± 9.08	42.64 ± 8.78	-	0.618	0.537
Total scores	83.83 ± 17.40	85.41 ± 17.54	-	0.623	0.534
Mean FD (mm)	0.060 ± 0.041	0.063 ± 0.056	0.071 ± 0.048	1.611	0.201

Sex differences among AVH, NAVH and HC groups were examined by Chi-square tests. Differences of continuous variables among AVH, NAVH and HC groups were examined by one-way analyses of variance (ANOVA). The difference of PANSS scores between AVH and NAVH groups was examined by two sample T-tests. AVH, schizophrenia patients with auditory verbal hallucination; NAVH, schizophrenia patients without auditory verbal hallucination; HC, healthy control; FD, framewise displacement; PNASS, Positive and Negative Syndrome Scale; AHRS, Auditory Hallucination Rating Scale; SD, standard deviation.

### Differences in dynamic functional connectivity

#### Auditory association cortex (AAC)

**Figure 1:**
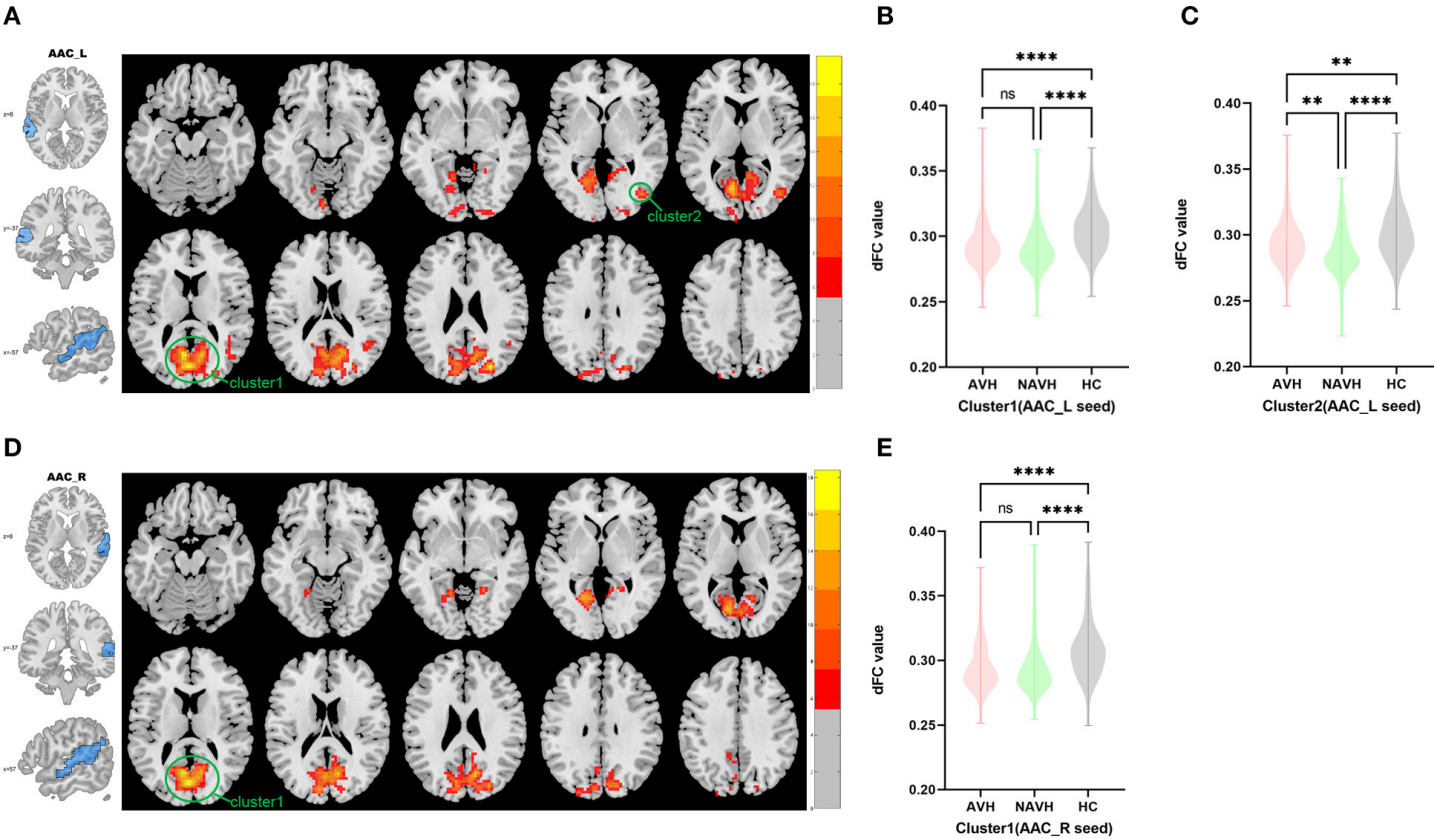
Brain regions showing abnormal dFC values among AVH, NAVH and HC groups in MNI space using left and right AAC as seeds. **(A)** Significant dFC value differences were observed in cluster1 (bilateral calcarine gyrus, bilateral cuneus gyrus, bilateral lingual gyrus, bilateral superior occipital gyrus, bilateral precuneus gyrus, posterior cingulate gyrus) and cluster2 (right middle temporal gyrus and right middle occipital gyrus) using left AAC as seed. **(B,C)** Altered dFC values in cluster1 and cluster2 among AVH, NAVH and HC groups using left AAC as seed. **(D)** Significant dFC value differences were observed in cluster1 (bilateral calcarine gyrus, posterior cingulate gyrus, bilateral cuneus gyrus, bilateral lingual gyrus, bilateral superior occipital gyrus, bilateral precuneus gyrus) using right AAC as seed. **(E)** Altered dFC values in cluster1 among AVH, NAVH and HC groups using right AAC as seed. ^****^Significant at 0.0001 level, ^***^significant at 0.001 level, ^**^significant at 0.01 level and ^*^significant at 0.05 level (2-tailed).

Among AVH, NAVH and HC groups, two clusters exhibiting statistical differences of dFC were showed using left AAC as seed. In *post hoc* statistical test, the AVH group showed no significant difference between the left ACC and cluster1, and increased dFC between the left ACC and cluster2 was observed in the AVH group (see Figure 1, Table 2).

**Table 2:** Whole-brain seed-based dynamic functional connectivity analysis results.

**ROI**	**ANOVA results**	**AVH** ** vs** ** NAVH**	**Brain areas(L/R)**	**Cluster size (voxels)**	**Peak coordinates (MNI)**	***T*-value**
					**x y z**	
**AAC_L**	Cluster1	/	calcarine gyrus (L/R), cuneus gyrus (L/R), lingual gyrus (L/R), superior occipital gyrus (L/R), precuneus gyrus (L/R), posterior cingulate gyrus	1244	27–78 24	19.67
	Cluster2	+	middle temporal gyrus (R) and middle occipital gyrus (R)	118	42–72 3	12.15
**AAC_R**	Cluster1	/	calcarine gyrus(L/R), posterior cingulate gyrus, cuneus gyrus (L/R), lingual gyrus (L/R), superior occipital gyrus (L/R), precuneus gyrus (L/R)	1077	−9–66 12	18.42
**HES_L**	Cluster1	–	superior occipital gyrus (L), cuneus gyrus (L), precuneus gyrus (L)	130	−21 42–21	10.81
	Cluster2	/	anterior orbitofrontal gyrus (L)	155	−15–84 30	10.22
**HES_R**	Cluster1	/	calcarine gyrus (L)	36	−18–66 12	8.87
	Cluster2	–	posterior cingulate gyrus	58	−3–30 30	10.43
**MGN_L**	Cluster1	–	calcarine gyrus (L/R), cuneus gyrus (L/R), lingual gyrus (L/R)	434	15–78 27	12.49
**MGN_R**	Cluster1	/	calcarine gyrus (L/R), cuneus gyrus(L/R), lingual gyrus (L/R)	191	−12–60 3	11.63
	Cluster2	/	precentral gyrus (L) and postcentral gyrus (L)	50	−36–15 63	8.70

MNI, Montreal Neurological Institute; ROI, region of interest; AAC, auditory association cortex; HES, Heschl's gyrus; MGN, medial geniculate nucleus; R, right; L, left; +, positive; –, negative; /, no significant difference.

Among AVH, NAVH and HC groups, one cluster exhibiting statistical differences of dFC were showed using the right AAC as seed. In *post hoc* statistical test, no difference was found between right ACC and cluster1 in the AVH group (see Figure 1, Table 2).

#### Heschl's gyrus (HES)

**Figure 2:**
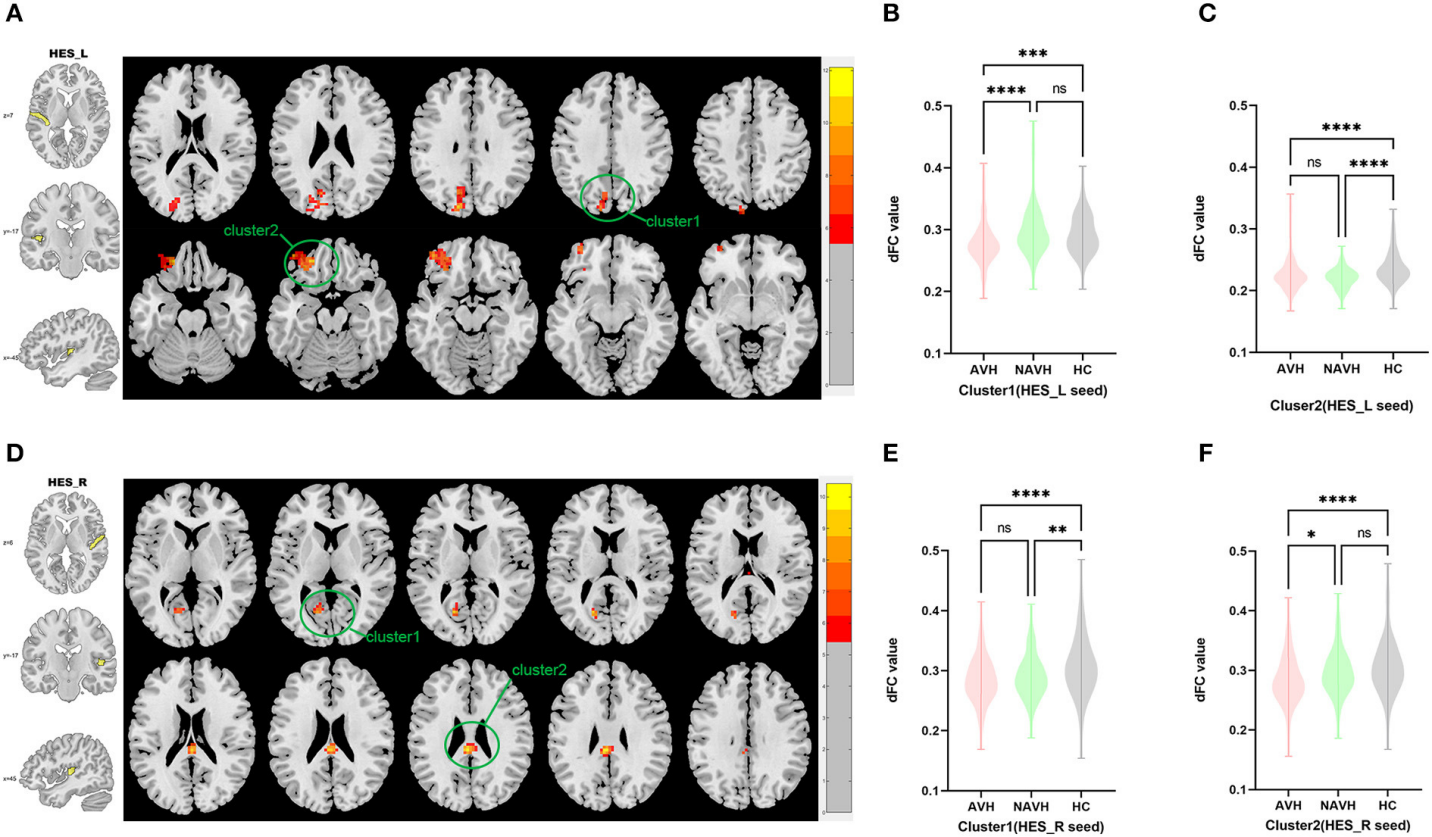
Brain regions showing abnormal dFC values among AVH, NAVH and HC groups in MNI space using left and right HES as seeds. **(A)** Significant dFC value differences were observed in cluster1 (left superior occipital gyrus, left cuneus gyrus, left precuneus gyrus) and cluster2 (left anterior orbitofrontal gyrus) using left HES as seed. **(B,C)** Altered dFC values in cluster1 and cluster2 among AVH, NAVH and HC groups using left HES as seed. **(D)** Significant dFC value differences were observed in cluster1 (left calcarine gyrus) and cluster2 (posterior cingulate gyrus.) using right HES as seed. **(E,F)** Altered dFC values in cluster1 among AVH, NAVH and HC groups using right HES as seed. ****Significant at 0.0001 level, ***significant at 0.001 level, **significant at 0.01 level and *significant at 0.05 level (2-tailed).

Among AVH, NAVH and HC groups, two clusters exhibiting statistical differences of dFC were showed using left HES as seed. In *post hoc* statistical test, decreased dFC between the left HES and cluster1was observed in the AVH group, and no difference was found between left HES and cluster2 in the AVH group (see Figure 2, Table 2).

Among AVH, NAVH and HC groups, two clusters exhibiting statistical differences of dFC were showed using right HES as seed. In *post hoc* statistical test, no difference was found between right HES and cluster1 in the AVH group, and decreased dFC between the right HES and cluster2 was observed in the AVH group (see Figure 2, Table 2).

#### Medial geniculate nucleus (MGN)

**Figure 3:**
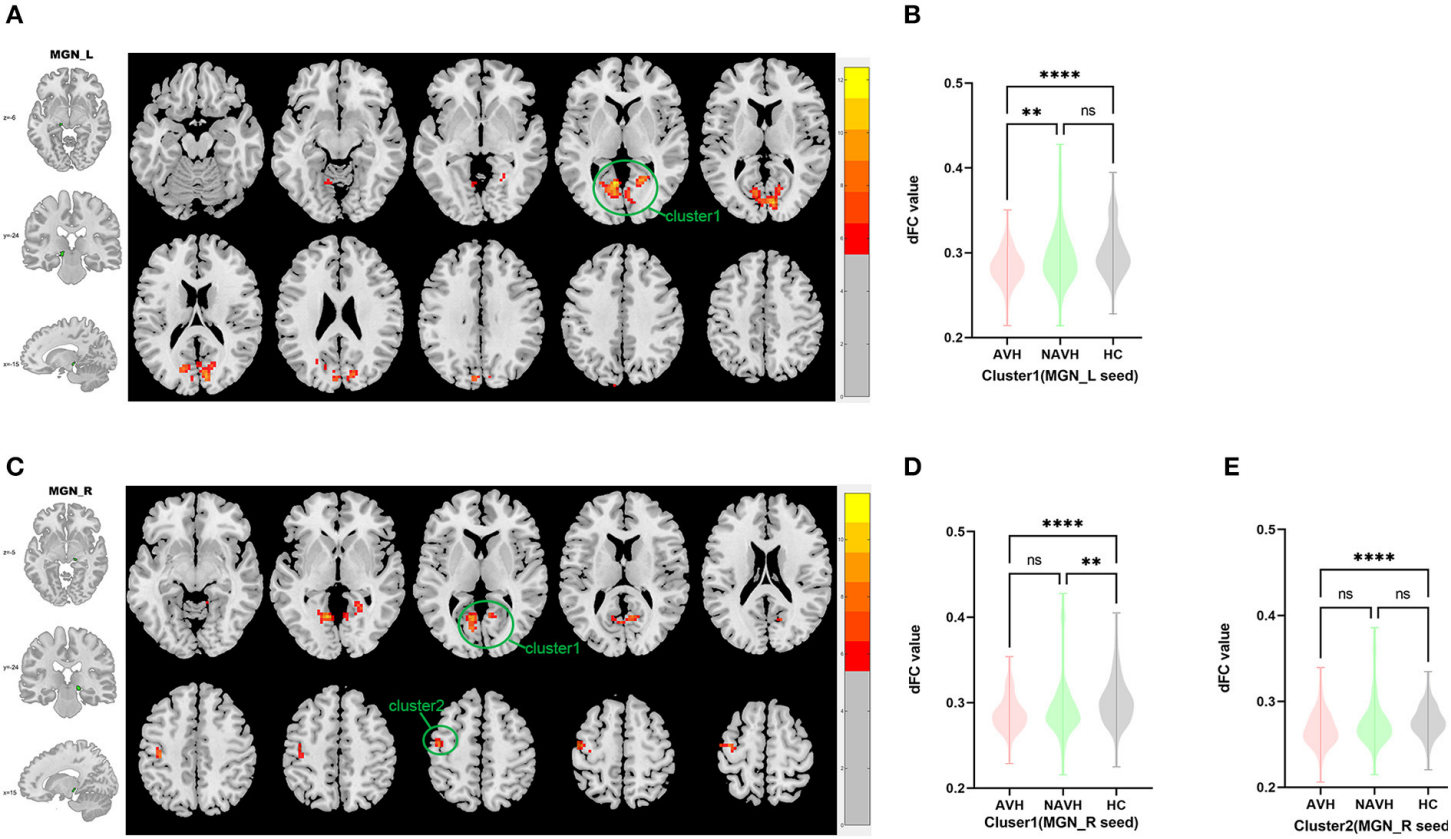
Brain regions showing abnormal dFC values among AVH, NAVH and HC groups in MNI space using left and right MGN as seeds. **(A)** Significant dFC value differences were observed in cluster1 (bilateral calcarine gyrus, bilateral cuneus gyrus, bilateral lingual gyrus) using left MGN as seed. **(B,C)** Altered dFC values in cluster1 among AVH, NAVH and HC groups using left MGN as seed. **(D)** Significant dFC value differences were observed in cluster1 (bilateral calcarine gyrus, bilateral cuneus gyrus, bilateral lingual gyrus) and cluster2 (left precentral gyrus and left postcentral gyrus) using right MGN as seed. **(E,F)** Altered dFC values in cluster1 among AVH, NAVH and HC groups using right MGN as seed. ****Significant at 0.0001 level.

Among AVH, NAVH and HC groups, one cluster exhibiting statistical differences of dFC was showed using left MGN as seed. In *post hoc* statistical test, decreased dFC between the left MGN and cluster1 was observed in the AVH group (see Figure 3, Table 2).

Among AVH, NAVH and HC groups, two clusters exhibiting statistical differences of dFC was showed using right HES as seed. In *post hoc* statistical test, no difference was found between right MGN and cluster1/cluster2 in the AVH group (see Figure 3, Table 2).

### Spearman correlational analysis

**Figure 4:**
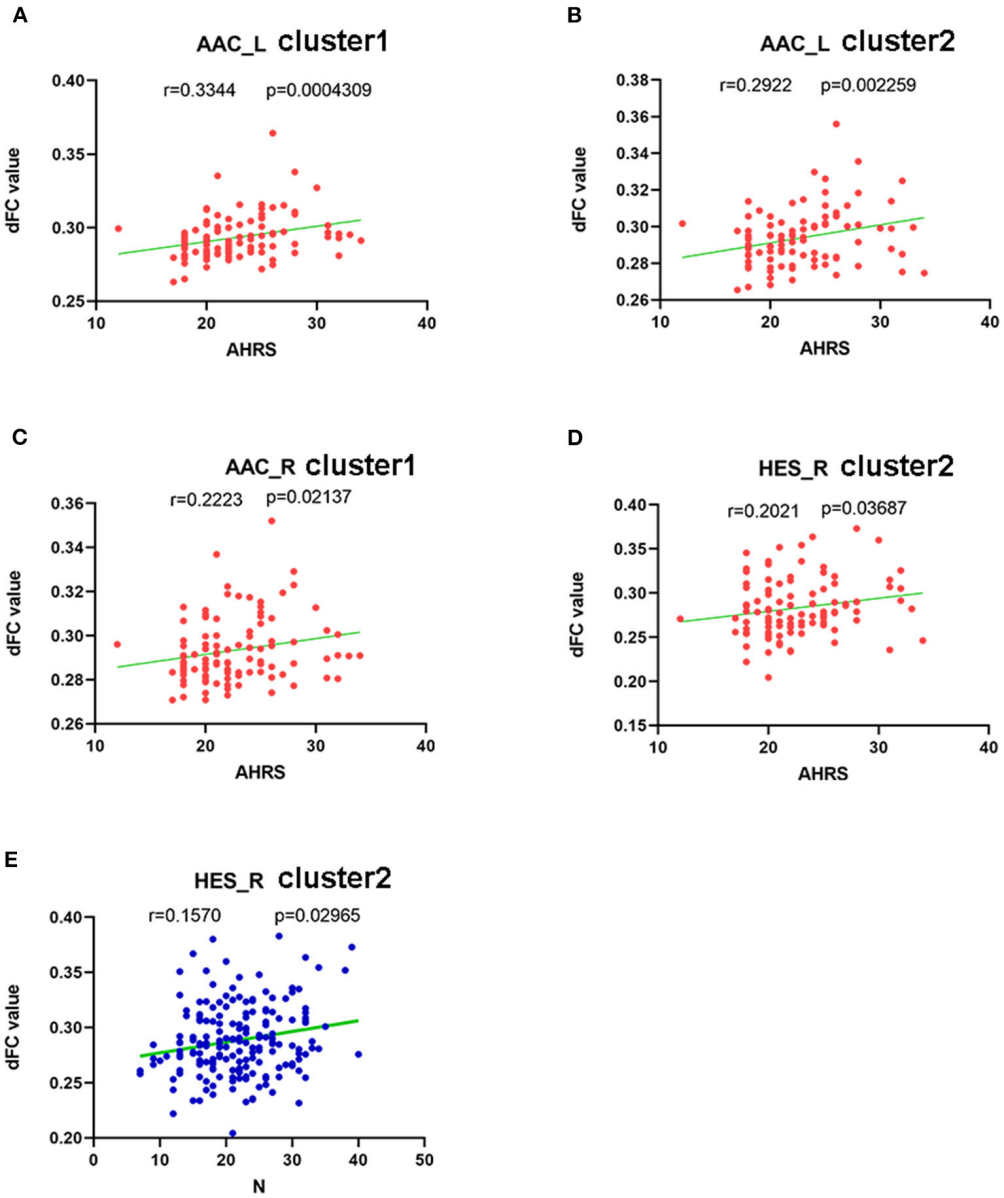
Correlations between abnormal dFC values and clinical variables. In schizophrenia patients with AVH. **(A)** a positive correlation was observed between the dFC values in the cluster1 using left AAC as seed and AHRS. **(B)** a positive correlation was observed between the dFC values in the cluster2 using left AAC as seed and AHRS. **(C)** a positive correlation was observed between the dFC values in the cluster1 using rihgt AAC as seed and AHRS, and **(D)** a positive correlation was observed between the dFC values in the cluster2 using right HES as seed and AHRS. In schizophrenia patients with AVH and NAVH. **(E)** a positive correlation was observed between the dFC values in the cluster2 using right HES as seed and PANSS negative sub-scores.

The AHRS scores were significantly positively correlated with the dFC values of cluster 1 using left AAC seed (*r* = 0.334, *p* = 0.000), cluster 2 using left AAC seed (*r* = 0.292, *p* = 0.002), cluster 1using right AAC seed (*r* = 0.222, *p* = 0.021) and cluster 2 using right HES seed (*r* = 0.202, *p* = 0.037) in the AVH group (see Figure 4).

PANSS negative sub-scores were negatively correlated with the dFC values of cluster 2 using the right HES seed in both AVH and NAVH groups (*r* = 0.157, *p* = 0.029) (see Figure 4).

There is no significance between dFC values of all above regions and PANSS total scores, PANSS positive, and general sub-scores.

### Validation results

To validate our core dFC conclusions, we tested two alternative sliding window widths. The sliding window lengths of 50 and 60 TRs produced alike outcomes to the 30 TR in our study. All validation analysis results were presented as Supplementary material.

## Discussion

Our study investigated the whole-brain dFC using auditory-related regions as seeds in first-episode, drug- naïve schizophrenia patients with and without AVH compared to healthy controls. In our study, the AVH group showed significantly increased dFC between left ACC and right middle temporal gyrus and right middle occipital gyrus, decreased dFC between left HES and left superior occipital gyrus, left cuneus gyrus, left precuneus gyrus, decreased dFC between right HES and posterior cingulate gyrus, and decreased dFC between left MGN and bilateral calcarine gyrus, bilateral cuneus gyrus, and bilateral lingual gyrus. Moreover, we found positive correlations between the AHRS and the dFC of significantly brain areas using left ACC, right ACC, and right HES as seeds respectively. Together, these results suggest that auditory related areas in schizophrenia patients present abnormal dynamic connectivity with multiple brain areas including the middle temporal gyrus, middle occipital gyrus, superior occipital gyrus, cuneus gyrus, precuneus gyrus, calcarine gyrus, cuneus gyrus, lingual gyrus, and posterior cingulate gyrus, which act a crucial part in the mechanism of AVH.

Taking the brain area related to auditory perception as the node and the relationship between the two nodes as the edge, the sum of these nodes and edges is called the auditory network, also known as the auditory pathway. Beginning with the primary auditory cortex, there are two auditory pathways—ventral and dorsal pathway (25, 26), which correspond to different specific functions in auditory information processing. Sound is first received and initially processed though the primary auditory cortex, afterwards transmitted to the secondary auditory cortex (located in BA22 and BA42) for processing, and then transferred to the higher information processing brain areas through the thalamus. Several research results speculate that the multiple auditory functions of the AVH group are abnormal, whether it is primary auditory processing or advanced language processing.

MGN is left-right symmetrical and located in the posterior medial part of the thalamus, which is an important relay station in the auditory pathway. The medial geniculate body can be segmented into ventral, dorsal, and central part. The ventral region and dorsal region can project to the auditory cortex through the thalamic cortical bundle, while the central region can also project to the non-auditory cortex (43). The central and dorsal regions involve the interaction of multiple senses and the interaction of auditory signals with other signals (44). In a word, MGN is one of the regions to complete the interaction between auditory and somatosensory signals. According to previous studies, auditory and somatosensory signals will be transmitted to MGN and then transmitted to other cortex after processing (45), so we believe that the symptoms of verbal auditory hallucination may be related to the abnormal connection between the medial geniculate body and other brain regions. Our study found that the abnormal connections between the medial geniculate body and calcarine gyrus, cuneus gyrus, lingual gyrus, central anterior gyrus, and central posterior gyrus were related to auditory hallucination, although no significant correlation was found between these abnormal connections and AHRS.

The majority of fMRI studies on the mechanism of AVH in schizophrenia have concentrated on inherent connectivity of areas relating to auditory, and language processing, involving the frontal and temporal lobe. It is known to all that Broca's and Wernicke's areas act a crucial part in speech processing, Hoffman and many other researchers have observed increased connectivity between the left inferior frontal gyrus (IFG) and STG, which is a unique pattern of auditory hallucination in schizophrenia patients (46). According to the study reported by Shinn et al., compared with patients without AVH, schizophrenic patients with AVH presented increased FC between left HES and left frontal-parietal region, which is relevant to the severity of AVH (47). Moreover, the pre-frontal cortex is also one of 25 nodes in the AVH network revealed by Scheinost et al. (48). Our study found the abnormal functional connectivity between left HES and left anterior orbito-frontal gyrus among AVH, NAVH, and HC groups, with the mean dFC value in the AVH group higher than NAVH group, although the difference was not statistically significant. Combined with the results of this study, we found abnormal fronto-temporal dFC in both the AVH group and NAVH group compared with the HC group, which may reveal that the abnormal fronto-temporal functional connectivity is not only related to the mechanism of AVH in schizophrenia, but also the pathogenesis of schizophrenia. While the dFC value of AVH group is higher than that of NAVH group, which may indicate that the abnormal fronto-temporal functional connectivity plays a greater role in schizophrenia patients with AVH than NAVH. Thus, to some extent, our results reflect the role of abnormal fronto-temporal FC in the occurrence of AVH.

The middle temporal gyrus is located between the superior temporal sulcus and the inferior temporal sulcus. There are numerous studies showing that the middle temporal gyrus is related to language-related activities and processes (49, 50), such as semantic processing, semantic cognition (51), word generation (52), vocabulary integration (53), sentence understanding, and complex sound processing. More and more studies observed structural and functional changes of the middle temporal gyrus which are significantly related to schizophrenia (54–57). Further, some researchers have found that AVH in patients with schizophrenia are related to the middle temporal gyrus (58, 59). Therefore, it is reasonable to believe that the abnormal FC between the middle temporal gyrus and auditory-related brain areas may be one of the mechanisms of auditory hallucination, which is also supported by the results of our study.

Less studies focus on the association between the occipital lobe and schizophrenia. Tohid et al. reviewed several studies (60) and discovered that schizophrenia is related to the abnormal structural and functional changes of the occipital lobe. The occipital lobe abnormalities were reported to act a crucial part in the mechanism of visual hallucination in schizophrenia. Up to now, few studies have found that the occipital lobe is related to auditory hallucination. Our data revealed that aberrant occipital-temporal and occipital-MGN dynamic FC may be associated with the mechanism of AVH in schizophrenia patients. More and more studies observed that the occipital lobe is associated with phonological and semantic modulation (61, 62), thus we speculate that abnormal conduction between the occipital lobe and auditory pathway may lead to aberrant phonological or semantic processing, which may lead to AVH in schizophrenia patients. Moreover, a “multisensory interplay” theory (63, 64) may be another corroboration of our results, Lewis et al. also reported that audio visual synchronization combines dynamic visual and aural inputs to create a more powerful and dependable multiple sensory system (65). Doehrmann et al. (66) revealed the sensory integrative effects of non-primary auditory and extra striate visual cortices by fMRI method. Eckert et al. (67) suggests that there is an obvious functional relevance between the primary auditory cortex and the anterior visual cortex. Thoma et al. (68) discovered that the occipital-temporal network showed significant AVH-related profiles, especially during AVH-off periods. Combined with the importance of connectivity between the auditory and visual cortex and the performance of audio-visual functional integration in the above studies, our results may suggest that abnormal occipitotemporal FC act a crucial part in the mechanism of AVH in schizophrenia patients.

Our study also observed abnormal dFC between the temporal lobe and posterior cingulate gyrus/precuneus, part of the DMN. Several articles have reported particular links between DMN and auditory hallucinations. Northoff et al. (14) and Jardri et al. (69) observed a chaotic situation concerning resting-state activity and external stimulation produced by coactions between the auditory cortex and parts of the DMN, which lead to auditory hallucinations eventually. Scheinost et al. (48) discovered a prospective AVH network by data-driven analysis of functional connectivity consisting of 25 nodes, including the medial pre-frontal cortex and posterior cingulate cortex, and revealing generous overlap with the DMN and language network.

Conventional sFC represents average connectivity strength between brain regions, while dFC reflects functional connectivity variability over temporal scales as a complementary method (31). Yet, there is increasing evidence that communication between different regions is not static but dynamic during the whole resting state scanning process, which is due to the conditional dependence of neural activity. Temporal variations in connectivity strength can't be captured by sFC. Consequently, the observation that the connections between brain regions increase or decrease over time is helpful to further understand the neural mechanism of AVH in schizophrenia from the perspective of temporal stability. In our study, dFC method was used to discover the variability of the functional connectivity over time between auditory related regions and other regions of the whole brain, which explain the role of auditory-related regions' connectivity stability in the mechanism of auditory hallucination.

Few potential limitations ought to be noticed in present study. First, the sample capacity of this study is not sufficient, so in the future, larger sample size is required to enhance the generalizability of the present findings. Second, patients in AVH group may suffer hallucinations or doze off in the process of scanning, which may disturb resting state design. Third, our study just obtained resting-state fMRI scans, and follow-up study ought to be regarded in the future. Fourth, we didn't evaluate further details on the severity of AVH, such as the incidence rate, content, and pain degree of auditory hallucinations. Future research needs to quantify the symptoms of auditory hallucinations in more detail. Consequently, our results ought to be regarded as initial, and further longitudinal studies with larger sample sizes should be investigated to formulate final conclusions.

## Conclusion

Our study used the dFC analysis to explore the abnormal dynamic functional connectivity of auditory-related regions in whole-brain areas in schizophrenia patients with AVH. We also correlated dFC values with PANSS and AHRS scores. The current results showed abnormal dynamic functional connectivity between the occipital lobe, middle temporal gyrus, posterior cingulate gyrus, precuneus gyrus, and cuneus gyrus with auditory-related regions, which is related to AVH. Taken together, the present findings demonstrate that schizophrenia patients with AVH showed multiple abnormal dFC brain regions using auditory related cortex and nucleus as seeds, suggesting that the distinct dFC patterns of auditory related regions may provide a potential neural mechanism of AVH in schizophrenia.

## Data availability statement

The original contributions presented in the study are included in the article/Supplementary material, further inquiries can be directed to the corresponding author.

## Ethics statement

The studies involving human participants were reviewed and approved by the Ethics Committee of the First Affiliated Hospital of Zhengzhou University. Written informed consent to participate in this study was provided by the participants' legal guardian/next of kin.

## Author contributions

KX contributed to the conception and design of this study. JC and YW recruited the participants and performed the MRI examination. KX, SH, and CW performed data processing. YC performed the statistical analysis. KX wrote the first draft of the manuscript. YZ, XS, and JC provided critical revision of the manuscript. All authors contributed to the article and approved the submitted manuscript.

## Funding

This work was supported by the Medical science and technology research project of Henan province (SBGJ202101013) and Medical Science and Technology Research Project of Henan Province (201701011).

## Conflict of interest

The authors declare that the research was conducted in the absence of any commercial or financial relationships that could be construed as a potential conflict of interest.

## Publisher's note

All claims expressed in this article are solely those of the authors and do not necessarily represent those of their affiliated organizations, or those of the publisher, the editors and the reviewers. Any product that may be evaluated in this article, or claim that may be made by its manufacturer, is not guaranteed or endorsed by the publisher.
