# Supplementary material for: Altered dynamic functional connectivity of auditory cortex and medial geniculate nucleus in first-episode, drug-naïve schizophrenia patients with and without auditory verbal hallucinations
Source: Front Psychiatry. 2022 Sep 7;13:963634. doi: 10.3389/fpsyt.2022.963634 (PMC9489854; doi:10.3389/fpsyt.2022.963634)
Supplement: Supplementary file 1 [file Data_Sheet_1.docx]

Supplementary Material

## Supplementary Figures

**
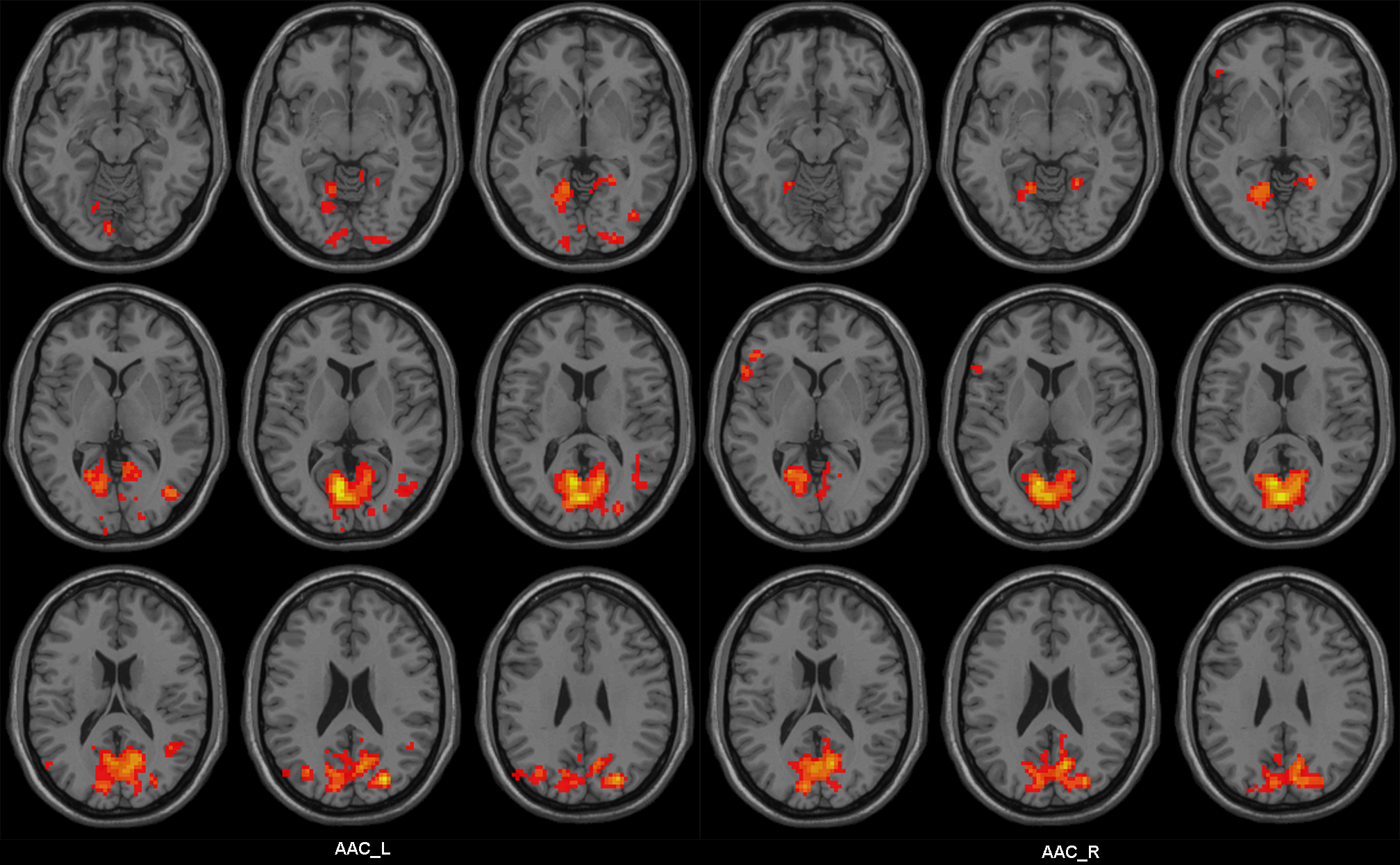
**

**Supplementary Figure 1.** Brain regions showing abnormal dFC values among AVH, NAVH and HC groups in MNI space using left and right AAC as seeds with the sliding window length of 50 TR. (Left part) Significant dFC value differences were observed in cluster1(bilateral calcarine gyrus, bilateral cuneus gyrus, bilateral lingual gyrus, bilateral superior occipital gyrus, bilateral precuneus gyrus, posterior cingulate gyrus) and cluster2(right middle temporal gyrus and right middle occipital gyrus) using left AAC as seed. (Right part) Significant dFC value differences were observed in cluster1(bilateral calcarine gyrus, posterior cingulate gyrus, bilateral cuneus gyrus, bilateral lingual gyrus, bilateral superior occipital gyrus, bilateral precuneus gyrus) and cluster2(left inferior frontal gyrus) using right AAC as seed.


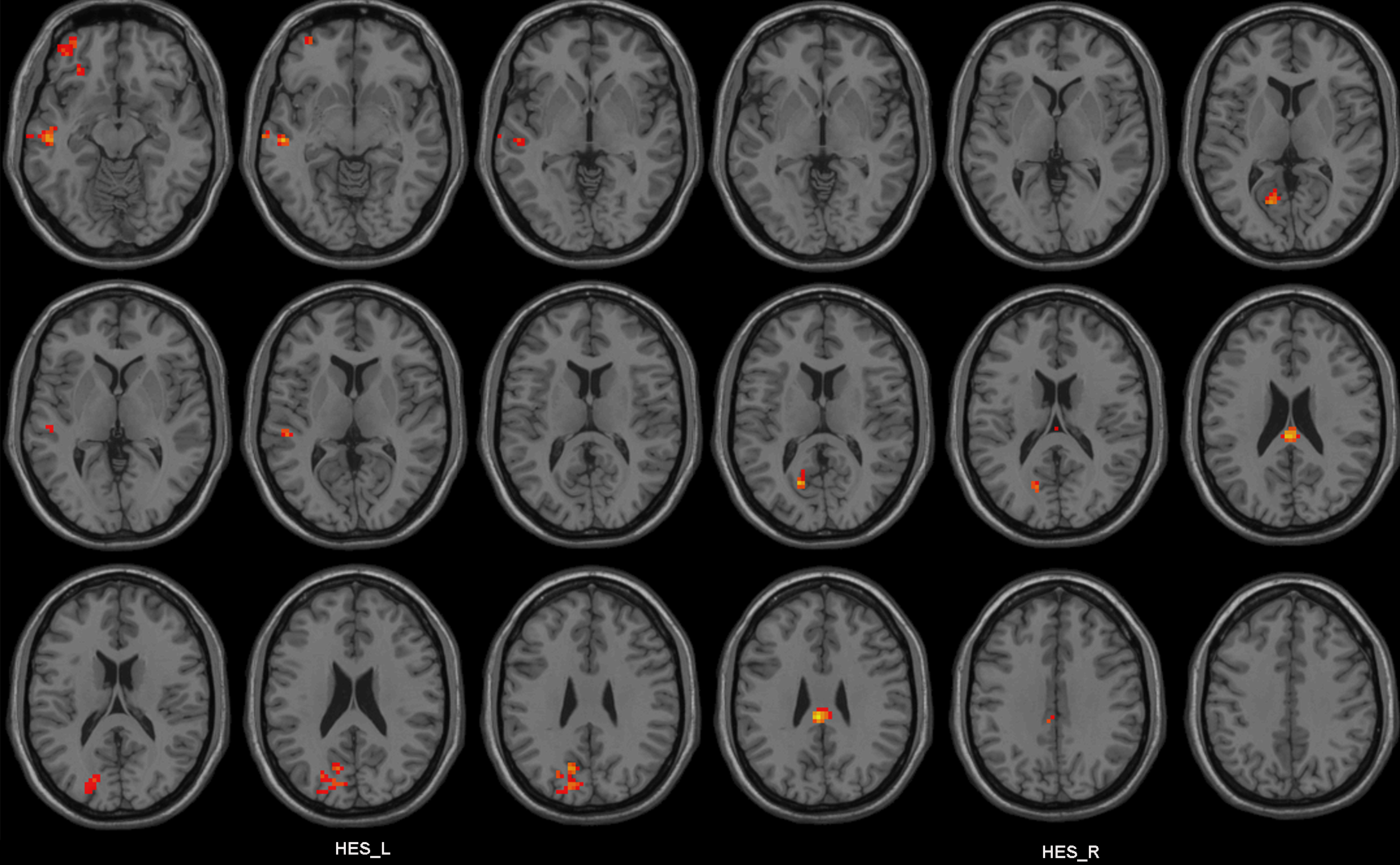


**Supplementary Figure 2.** Brain regions showing abnormal dFC values among AVH, NAVH and HC groups in MNI space using left and right HES as seeds with the sliding window length of 50 TR. (Left part) Significant dFC value differences were observed in cluster1(left superior occipital gyrus, left cuneus gyrus, left precuneus gyrus), cluster2(left anterior orbitofrontal gyrus) and cluster3(left middle temporal gyrus )using left HES as seed. (Right part) Significant dFC value differences were observed in cluster1(left calcarine gyrus) and cluster2(posterior cingulate gyrus) using right HES as seed.


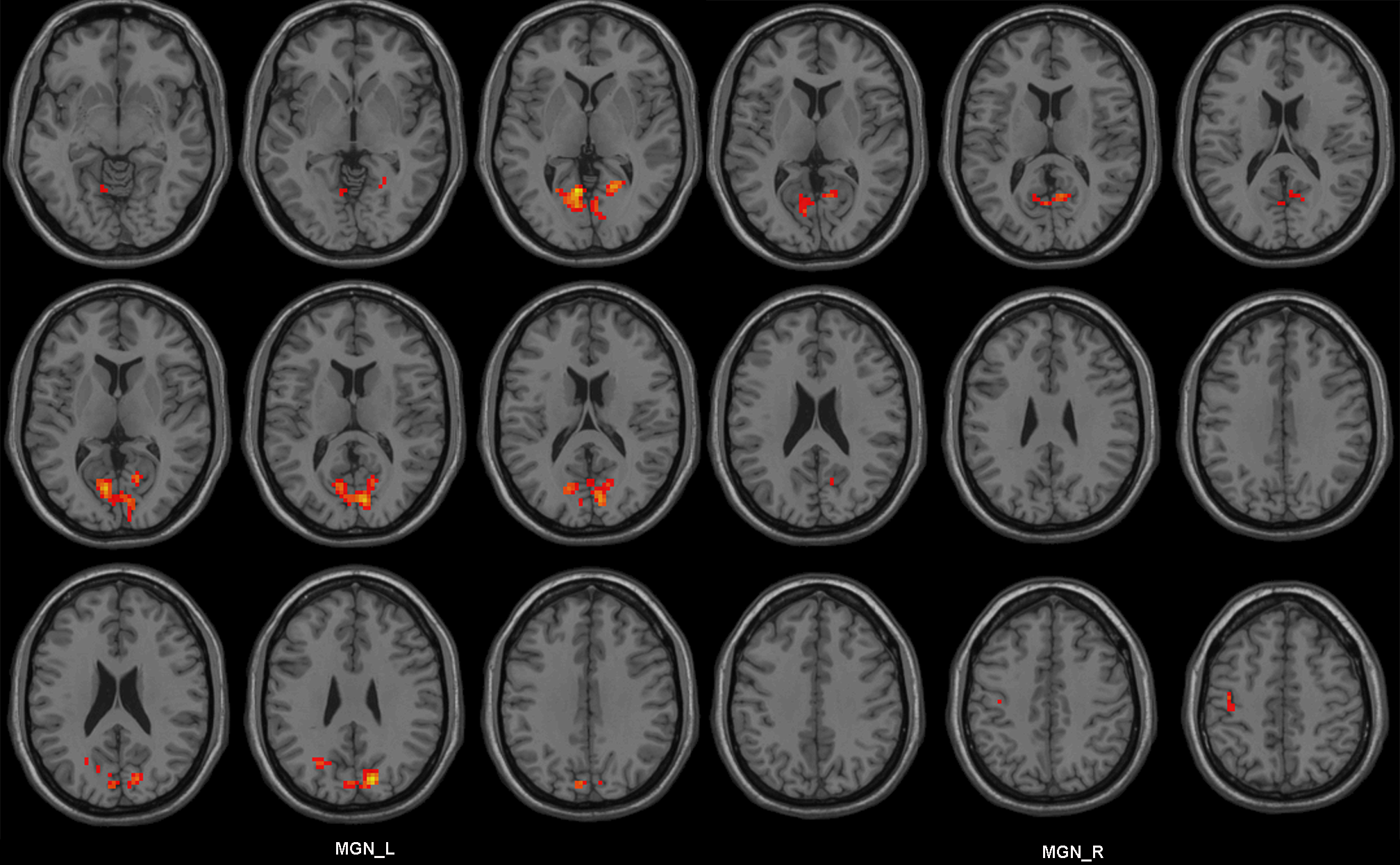


**Supplementary Figure 3.** Brain regions showing abnormal dFC values among AVH, NAVH and HC groups in MNI space using left and right MGN as seeds with the sliding window length of 50 TR. (Left part) Significant dFC value differences were observed in cluster1(bilateral calcarine gyrus, bilateral cuneus gyrus, bilateral lingual gyrus) using left MGN as seed. (Right part) Significant dFC value differences were observed in cluster1(bilateral calcarine gyrus, bilateral cuneus gyrus, bilateral lingual gyrus) and cluster2(left precentral gyrus and left postcentral gyrus) using right MGN as seed.


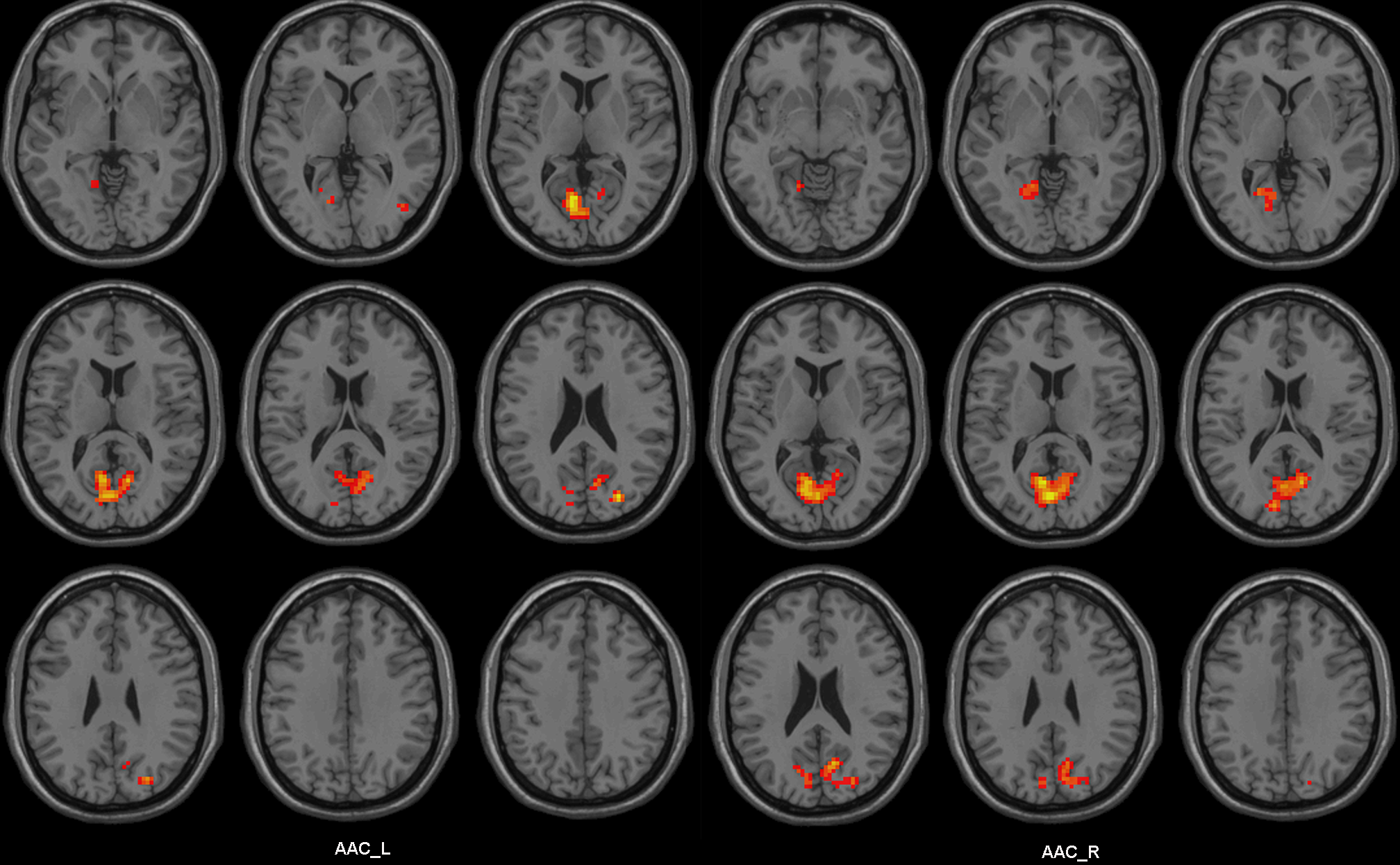


**Supplementary Figure 4.** Brain regions showing abnormal dFC values among AVH, NAVH and HC groups in MNI space using left and right AAC as seeds with the sliding window length of 60 TR. (Left part) Significant dFC value differences were observed in cluster1(bilateral calcarine gyrus, bilateral cuneus gyrus, bilateral lingual gyrus, bilateral superior occipital gyrus, bilateral precuneus gyrus, posterior cingulate gyrus) and cluster2(right middle temporal gyrus and right middle occipital gyrus) using left AAC as seed. (Right part) Significant dFC value differences were observed in cluster1(bilateral calcarine gyrus, posterior cingulate gyrus, bilateral cuneus gyrus, bilateral lingual gyrus, bilateral superior occipital gyrus, bilateral precuneus gyrus) using right AAC as seed.


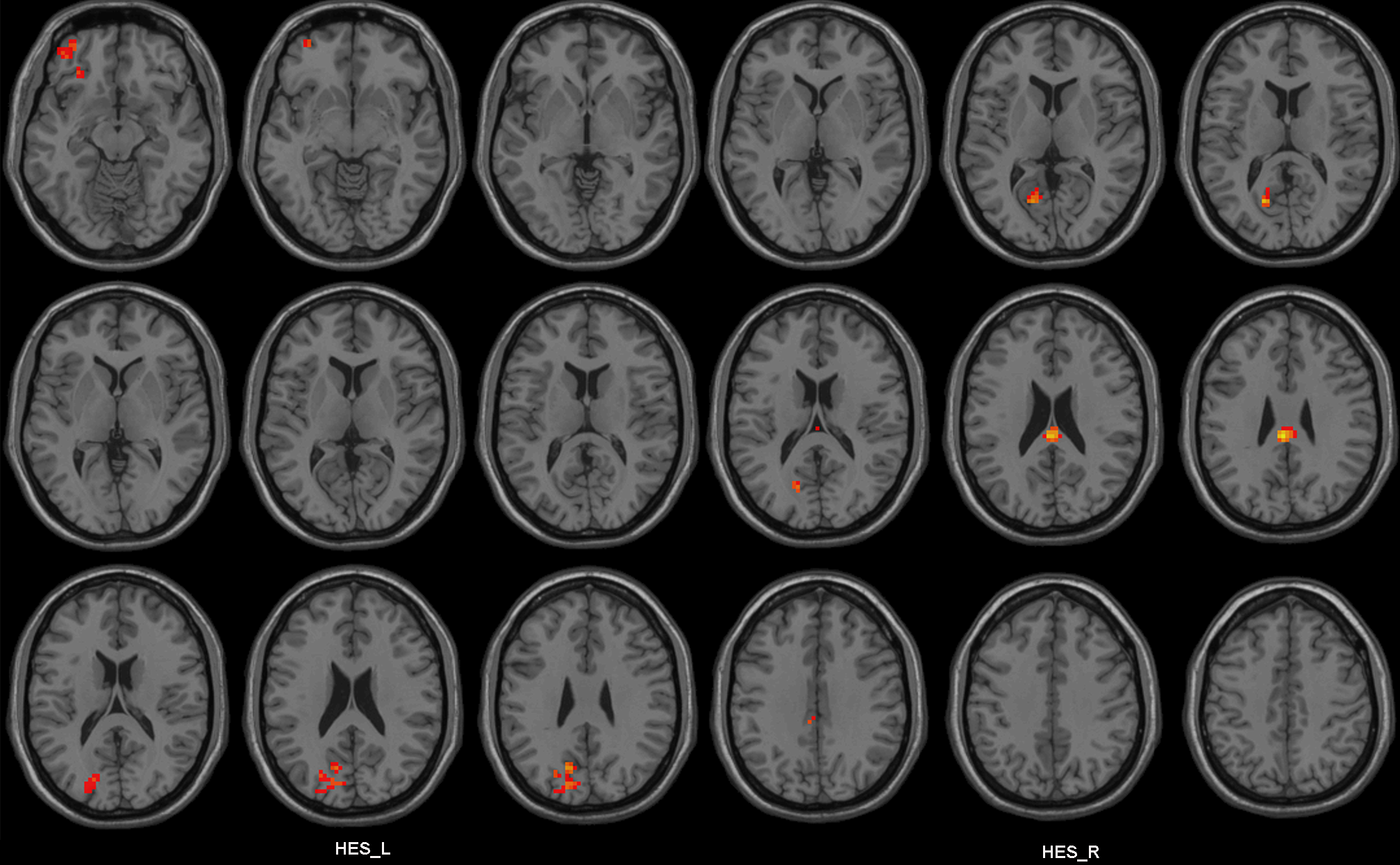


**Supplementary Figure 5.** Brain regions showing abnormal dFC values among AVH, NAVH and HC groups in MNI space using left and right HES as seeds with the sliding window length of 60 TR. (Left part) Significant dFC value differences were observed in cluster1(left superior occipital gyrus, left cuneus gyrus, left precuneus gyrus) and cluster2(left anterior orbitofrontal gyrus) using left HES as seed. (Right part) Significant dFC value differences were observed in cluster1(left calcarine gyrus) and cluster2(posterior cingulate gyrus) using right HES as seed.


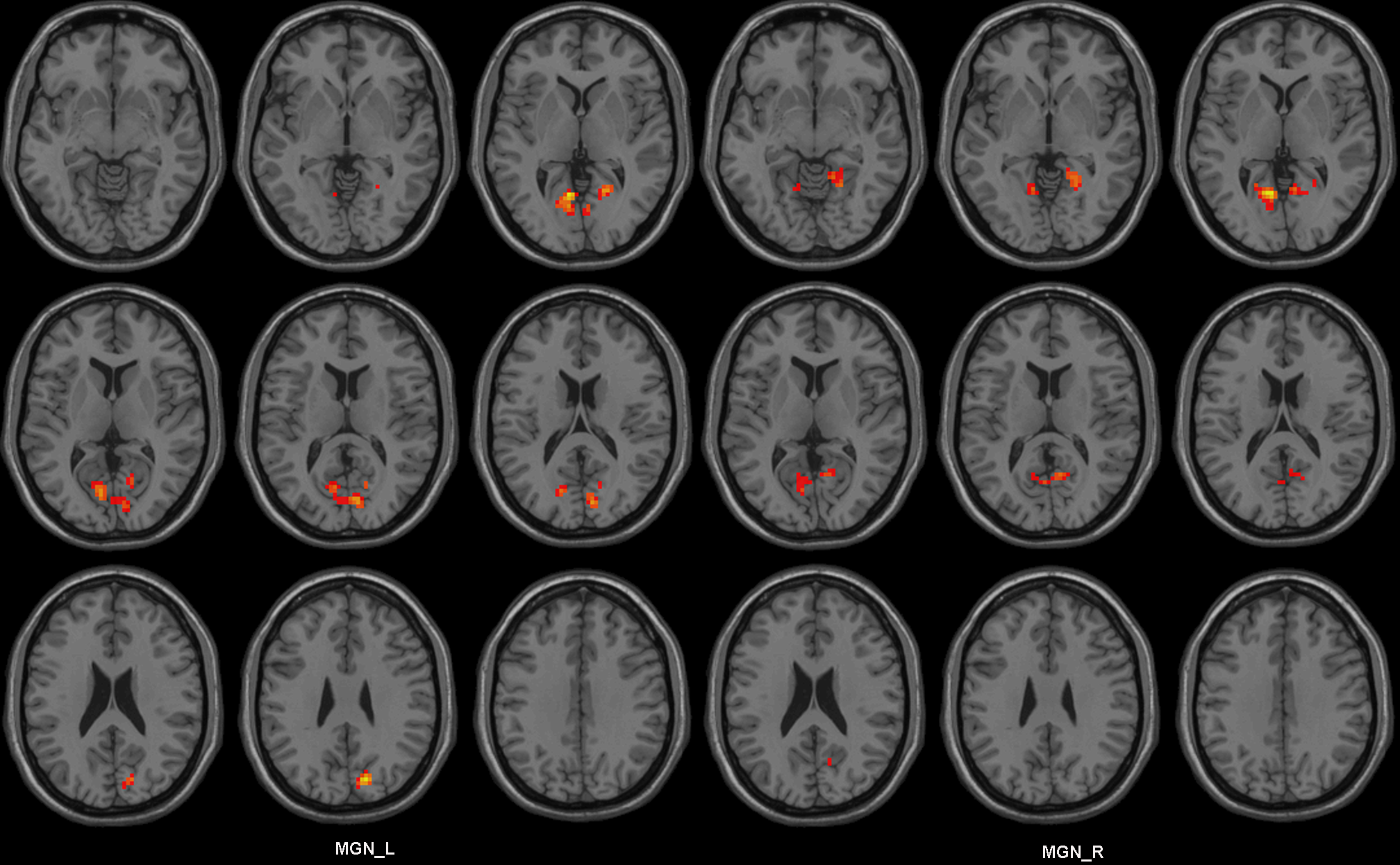


**Supplementary Figure 6.** Brain regions showing abnormal dFC values among AVH, NAVH and HC groups in MNI space using left and right MGN as seeds with the sliding window length of 60 TR. (Left part) Significant dFC value differences were observed in cluster1(bilateral calcarine gyrus, bilateral cuneus gyrus, bilateral lingual gyrus) using left MGN as seed. (Right part) Significant dFC value differences were observed in cluster1(bilateral calcarine gyrus, bilateral cuneus gyrus, bilateral lingual gyrus) using right MGN as seed.
